# Light-Triggered Sequence-Specific Cargo Release from DNA Block Copolymer-Lipid Vesicles**

**DOI:** 10.1002/anie.201206783

**Published:** 2012-10-26

**Authors:** Alberto Rodríguez-Pulido, Alina I Kondrachuk, Deepak K Prusty, Jia Gao, Maria A Loi, Andreas Herrmann

**Affiliations:** a Department of Polymer Chemistry, Zernike Institute for Advanced Materials, University of GroningenNijenborgh 4, 9747 AG Groningen (The Netherlands); b Department of Photophysics and Optoelectronics, University of Groningen(The Netherlands)

**Keywords:** Block copolymers, DNA, liposomes, nanocontainers, singlet oxygen

Nanocontainers have gained much importance because of their versatile properties and broad application potential in the fields of chemistry,^1^ biophysics,^2^ and nanomedicine.2b, 3 Lipid vesicles have proven to be a particularly effective class of nanocontainers, able to encapsulate and protect diverse small molecules, such as ions and drugs,^4^ as well as larger biomacromolecules, such as proteins or DNA.2a, 5 Moreover, the engineering of lipid vesicles has sufficiently advanced to a level which enables functionalization and manipulation of their surfaces with specific ligands to improve their poor chemical and physical specificity. For example, proteins (including antibodies),5a, 6 carbohydrates,^7^ and vitamins2a, 8 have all been used as targeting units anchored to the liposome surfaces to direct these nanocontainers to the site of action. More recently, single-stranded DNA covalently attached to cholesterol or lipid moieties has been incorporated into vesicle bilayers in order to exploit the specific recognition ability of oligonucleotides (ODNs) by hybridization with their complementary strands. These DNA hybrids have been shown to be critical building blocks in the construction of novel self-assembled supravesicular structures in which vesicles were linked by double-stranded ODNs,^9^ or utilized to induce programmed fusion.^10^ Moreover, DNA–lipids have been used to construct hybridization-sensitive nanocontainers,^11^ to improve liposome marking,^12^ to mimic cellular systems,^13^ and for multiplexed DNA detection.^14^ As demonstrated by the numerous examples above, the decoration of vesicles with DNA amphiphiles has resulted in significant advances in the functionality of these containers; the bilayer barrier itself remains a significant hindrance to the release of cargo, however. There have been several successful attempts to liberate cargo molecules from vesicles. One possibility is the generation of pores in the lipid bilayer through the incorporation of natural or synthetic ion channels.^15^ Another approach, which entails enzymes, makes use of selective lipases for cargo release.^16^ A promising alternative is the design of “smart” liposomes that are able to release cargo through physicochemical responses to external stimuli (such as nanoparticle incorporation into the membrane, or changes in pH or temperature).^17^ Furthermore, photosensitizers that generate singlet oxygen (^1^O_2_) upon light irradiation have been incorporated into the bilayer or the vesicle interior to mediate cargo release.^18^ Nevertheless, the liberation of cargo molecules from such functionalized nanocontainers is unfortunately not selective for mixed populations of vesicles and further work is needed to increase the specificity of these container systems. Herein, we report a powerful new approach for selective cargo release from lipid vesicles that is based on amphiphilic DNA block copolymers (DBCs) and the hybridization of photosensitizer units (Scheme 1). It was demonstrated that this new class of nucleic acid amphiphiles, DBCs, can be stably anchored in the phospholipid membrane of liposomes (step 1). The protruding ODN was functionalized with ODN-photosensitizer conjugates through Watson–Crick base pairing (step 2) and after light irradiation (step 3) selective cargo release was achieved (step 4) depending on the DNA code on the surface of the vesicles. DBCs, as used here for cargo release, consist of a single-stranded ODN covalently bound to an organic polymer block. The combination of highly specific DNA interactions with the hydrophobic properties of the polymer block make DBCs ideally suited to diverse nanoscience applications, for example, as gene and drug delivery systems, or as building blocks in nanoelectronic devices.^19^ Herein, we introduce a new application for DBCs: as a functionalization and release reagent for liposomes. DNA-*b*-polypropyleneoxide (DNA-*b*-PPO) was selected because of its amphiphilic nature, which leads the hydrophobic polymer segments to interact with the internal region of the lipid bilayer while the hydrophilic nucleotides remain on the liposome surface free to bind with the complementary DNA sequences. Additional features of DNA-*b*-PPO include its fully automated synthesis,^20^ the known ability of PPO to insert into the hydrophobic part of phospholipid bilayers,15d, 21 and its susceptibility to oxidation.^22^

**Scheme 1:**
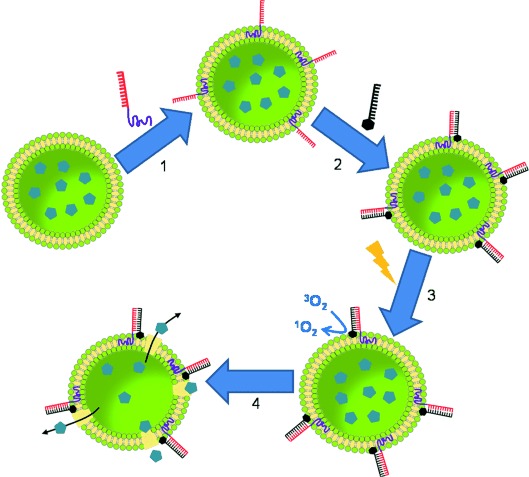
Illustration of selective cargo release from DBC-decorated lipid vesicles. 1) DBCs are stably anchored in unilamellar lipid vesicles; 2) DBC-decorated vesicles are functionalized with conjugated ODN-photosensitizers by hybridization; 3) singlet oxygen is generated by light irradiation; and 4) selective cargo release is induced by the oxidative effect of singlet oxygen.

The synthesis of DNA-*b*-PPO began with the phosphitylation of terminally hydroxy-functionalized PPO (number average molecular weight (*M*_n_)=1000 g mol^−1^) by 2-cyanoethyl *N*,*N*-diisopropylchlorophosphoramidite. The resulting activated polymer was coupled to the 5′ or 3′ end of a 22-mer ODN using standard or reverse solid-phase synthesis, respectively (for synthetic details and characterization of DNA-*b*-PPOs, see the Supporting Information). Table 1 summarizes the ODN sequences present in 22-*b*-PPO, complementary c22-*b*-PPO (conventional ODN synthesis), and r22-*b*-PPO (reverse ODN synthesis).

**Table 1:** Oligonucleotide (ODN) sequences present in DNA-*b*-PPO.

Entry	Abbreviation of ODN sequence	Sequence
1	22	PPO-5′-CCT CGC TCT GCT AAT CCT GTT A-3′
2	r22	5′-CCT CGC TCT GCT AAT CCT GTT A-3′-PPO
3	c22	PPO-5′-TAA CAG GAT TAG CAG AGC GAG G-3′

The ability to functionalize liposomes with DNA-*b*-PPO depends directly on its strong anchorage in the lipid membranes. To demonstrate such stable incorporation, a fluorescence resonance energy transfer (FRET) assay was carried out. The experiments were performed using phospholipid vesicles decorated with DBCs with a hydrodynamic diameter of [196±20] nm, as determined by dynamic light scattering analysis. They were created in Tris/HCl (10 mm, pH 7.4), NaCl buffer (150 mm; see the Supporting Information for further details). These liposomes, which consist of c22-*b*-PPO and 1,2-diphytanoyl-*sn*-glycero-3-phosphocholine (DPhyPC; lipid/DBC ratio: 370) were prepared in the presence of a lipid-modified acceptor dye lissamine rhodamine B 1,2-dihexadecanoyl-*sn*-glycero-3-phosphoethanolamine (N-Rh-PE). The donor, on the other hand, consisted of the dye Alexa 488 covalently attached to the 3′ end of a 22-mer ODN (r22-Alexa). Hybridization of r22-Alexa and c22-*b*-PPO incorporated in the fluorescent N-Rh-PE/DPhyPC liposomes brought the donor and acceptor close enough to each other at the surface (Figure 1 a), for an efficient FRET to be observed (Figure 1 b). Furthermore, the addition of Triton X-100 to this FRET system resulted in the disruption of the liposomes, and a consequent extension of the donor–acceptor distance, as indicated by the lower FRET efficiency and decreased acceptor emission. Moreover, Figure 1 b shows the spectra obtained in two controls (non-FRET systems; see the Supporting Information, Schemes S5 b and S5 c) in which r22-Alexa could not hybridize to the vesicle surface and thus did not undergo energy transfer, resulting in similar spectra to those observed after liposome disruption. These results confirm the incorporation of c22-*b*-PPO in the liposomes, with the DNA readily available for functionalization by Watson–Crick base pairing. With the aim of studying the time stability of the c22-*b*-PPO incorporation, the FRET system described above was mixed with pure DPhyPC liposomes at different v/v ratios (1:1, 1:10, and 1:100; see the Supporting Information, Scheme S6). Then the Alexa/Rhodamine fluorescence spectra were monitored in each mixture over 24 h (Figure 1 c). Assuming that DBCs diffuse from N-Rh-PE/DPhyPC liposomes to DPhyPC liposomes, a decrease in the acceptor band should be observed. However, the time plot in Figure 1 c shows that the acceptor/donor maximum intensity ratio *I*_590_/*I*_520_ is essentially invariable for each mixture and in fact remains at a similar value to that of the initial FRET system (see the Supporting Information for the detection limit of this method). These results confirm that the DBC is stably anchored in the lipid membrane over at least 24 h, which is promising for the use of this DNA amphiphile as a functionalizing reagent for liposomes.

**Figure 1:**
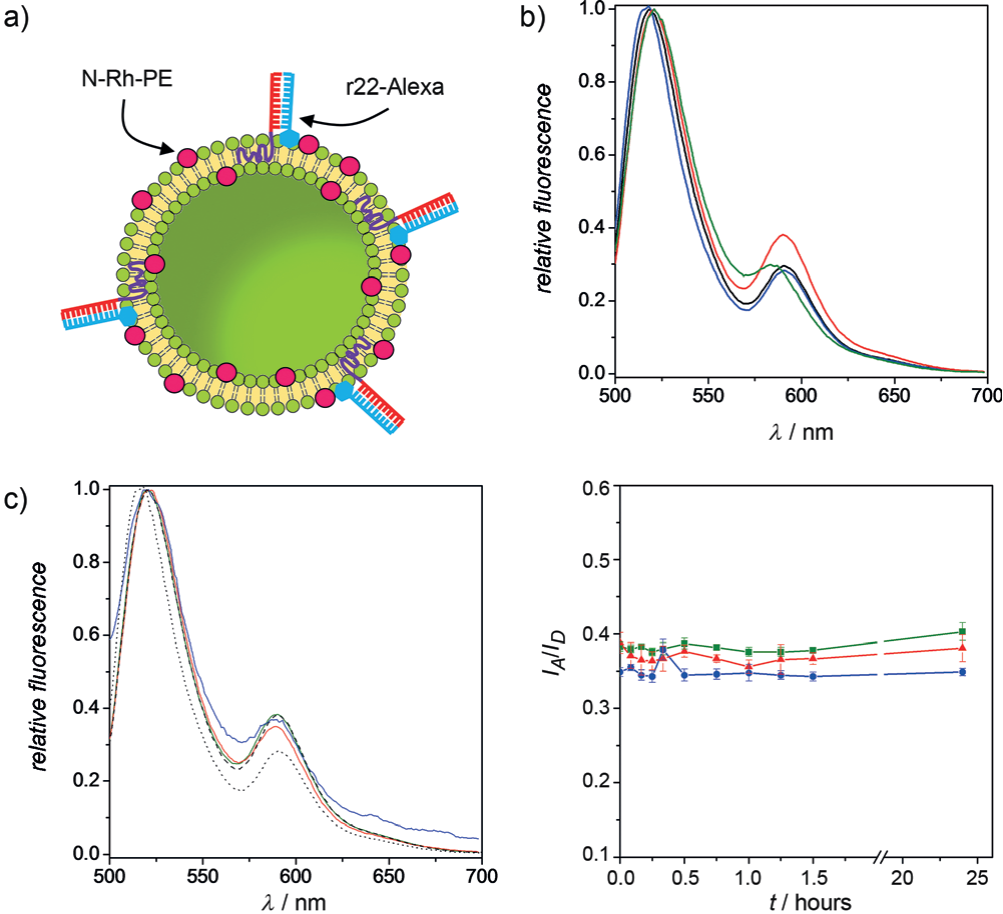
Study of the stable incorporation of DBCs in liposomes by a FRET assay. a) Illustration of the vesicles constituting the FRET-system: r22-Alexa (donor) is hybridized to c22-*b*-PPO/DPhyPC liposomes containing N-Rh-PE (acceptor). b) Fluorescence spectra of r22-Alexa/N-Rh-PE pair in FRET (red) and no-FRET (controls, blue and black) systems. Green line shows the spectrum of the FRET system after disrupting the liposomes with Triton X-100. c) Left: Fluorescence spectra of r22-Alexa/N-Rh-PE in the FRET system after mixing with pure DPhyPC liposomes at different v/v ratios: 1:1 (solid green line), 1:10 (solid red line) and 1:100 (solid blue line). Dotted and dashed lines represent the spectra of FRET and no-FRET systems, respectively, as controls without mixing with pristine vesicles. Right: Evolution of *I*_590_/*I*_520_ over time for the above three ratios.

Having confirmed the stable incorporation of DBCs at the liposome surface and thereby tagging the vesicles with a DNA code, we attempted to achieve light-induced sequence specific release (Scheme 1). A novel BODIPY monoiodine (BMI) photosensitizer was covalently attached to the 3′ end of a 22-mer ODN complementary to r22-*b*-PPO to create the sequence-specific ODN-photosensitizer c22-BMI (see the Supporting Information for synthetic details). The presence of a heavy atom on the BODIPY core favors inter-system crossing to the triplet state through increased spin-orbit interactions, thus transforming the fluorophore into a photosensitizing chromophore.^23^ In fact, measurements of the near-infrared ^1^O_2_ emission spectra have shown that BMI is a powerful photosensitizer, 14 times more efficient than the common standard Rose Bengal (Supporting Information, Figure S5). The functionalized nanocontainers were created by selectively hybridizing c22-BMI with r22-*b*-PPO/DPhyPC vesicles, loaded with calcein (100 mm). At that concentration the photoluminescence of this dye (Figure 2 a) is self-quenched, thus allowing the study of its release out of the containers by monitoring the increase in the fluorescence signal upon dilution of the chromophore in the surrounding environment.^24^ Subsequently, the vesicles were irradiated with light at a wavelength of 530 nm for 104 min to generate ^1^O_2_. As a consequence of this, the release of calcein to the surrounding medium was detected. Figure 2 b shows the proportion of calcein release from irradiated c22-BMI/r22-*b*-PPO/DPhyPC liposomes over time for several different lipid/DBC ratios of fully hybridized vesicles. The Figure also shows the release profiles of two controls: r22-*b*-PPO/DPhyPC liposomes in the absence of the photosensitizer, and c22-*b*-PPO/DPhyPC liposomes in the presence of c22-BMI, which cannot hybridize to the vesicles. The results summarized in Table 2 clearly show that the light-triggered cargo release increases with the amount of photosensitizer hybridized onto the vesicle surface, which reaches a maximum of 30 %, with much lower leakage observed in the controls. Although the oxidation of unsaturated lipids by ^1^O_2_ and its effect in membranes have been widely studied,18a–d no oxidative damage was reported for saturated lipids such as the DPhyPC used in this study. Therefore, it is assumed that the DBC plays an important role in the mechanism of release from the vesicles. In fact, we found degradation products when c22-BMI/r22-*b*-PPO hybrids were irradiated (Supporting Information, Figure S10). Although DNA was damaged by ^1^O_2_ to a considerable extent, it may not be the cause of the calcein release, owing to the fact that oxidation takes place on the vesicle surface. Instead, we anticipated that PPO is responsible for the destabilization of the membrane in our system. Although, to the best of our knowledge, PPO oxidation by ^1^O_2_ has not been reported, it is well established that ^1^O_2_ is involved in the formation of hydroxyl radicals, which have a high oxidative effect on the polymer.^22^ Indeed, an FT-IR spectroscopic analysis of the products formed from irradiating a mixture of BMI/PPO showed an increase in the carbonyl absorption peak at 1724 cm^−1^ when samples were exposed to light (Supporting Information, Figure S11). This result strongly supports that hydroxyl radicals and/or ^1^O_2_ are able to oxidize the PPO block inserted in the lipid membrane to such an extent that the polymer changes its polarity, which leads to destabilization of the bilayer and hence the release of calcein.

**Figure 2:**
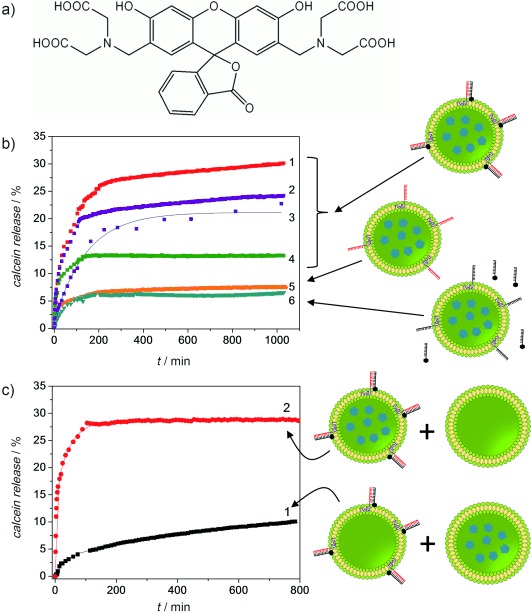
a) Chemical structure of the self-quenched fluorescence dye, calcein. b) Kinetics of cargo release from DBC-tagged vesicles induced by ^1^O_2_ formation: r22-*b*-PPO/DPhyPC liposomes hybridized with c22-BMI at different lipid/DBC ratios (curves 1–4: 1048, 2316, 5321, and 9271, respectively); r22-*b*-PPO/DPhyPC liposomes in the absence of c22-BMI (curve 5); and c22-*b*-PPO/DPhyPC liposomes in the presence of c22-BMI (curve 6). c) Light-induced sequence-specific release of calcein from a mixture of c22-BMI/r22-*b*-PPO/DPhyPC liposomes and calcein-loaded DPhyPC liposomes (curve 1), and a mixture of calcein loaded c22-BMI/r22-*b*-PPO/DPhyPC liposomes and non-loaded DPhyPC liposomes (curve 2). All samples were irradiated for 104 min.

**Table 2:** Calcein release from DBC–lipid liposomes induced by ^1^O_2_ formation.

Entry	DBC	Lipid/DBC	DBC/liposome[a]	Release [%]
1	r22-*b*-PPO	1048	274	30
2	r22-*b*-PPO	2316	124	24
3	r22-*b*-PPO	5321	54	21
4	r22-*b*-PPO	9271	31	13
5	c22-*b*-PPO	1048	274	7
6	r22-*b*-PPO^[b]^	1048	274	6

[a] Determined by Equation S3 in the Supporting Information. [b] Calcein release obtained in absence of photosensitizer.

Although the release mechanism cannot be unraveled in full detail, a strong correlation between DBC concentration in the bilayer and the degree of calcein release is evident. It should also be pointed out that calcein release primarily occurs during photo-excitation (Figure 2 b), whereas the liposomes change little after irradiation. The liposome damage must thus take place in the time in which ^1^O_2_ is generated and not afterwards, as expected from its short mean lifetime (*τ*=3.1 μs).^25^ The fact that all lipid/DBC ratios showed greater release than the controls demonstrates the importance of the proximity of c22-BMI and the lipid membrane for leakage to occur. Indeed, the mean diffusion length calculated for ^1^O_2_ generated in the bulk (ca. 157 nm) is one order of magnitude smaller than the average distance between contiguous vesicles (ca. 1860 nm), so it should only show high activity when generated close to the membrane where PPO is inserted (see the Supporting Information for calculation details). To support our hypothesis of sequence-specific cargo release, we designed a further experiment in which empty c22-BMI/r22-*b*-PPO/DPhyPC liposomes and pure DPhyPC liposomes loaded with calcein were mixed (50 % v/v) and irradiated (Figure 2 c). Although ^1^O_2_ generation took place at the surface of the DBC-lipid liposomes, the kinetic curve 1 of the figure only reflects the calcein release from nearby pure lipid vesicles, which were damaged as a consequence of the diffusion of the ^1^O_2_ in the buffer. The low yield of calcein release (ca. 8 %) is similar to previous control samples and contrasts with the high value obtained in the reverse sample (Figure 2 c, curve 2; pure DPhyPC liposomes mixed with calcein-loaded c22-BMI/r22-*b*-PPO/DPhyPC), which is similar to that obtained from loaded c22-BMI/r22-*b*-PPO/DPhyPC liposomes without mixing (ca. 30 %). This experiment shows that singlet oxygen has a negligible effect on non-targeted liposomes and thus confirms the sequence-specificity of our cargo release strategy.

For any future application, it would be desirable to increase the yield of targeted cargo release from such DBC-lipid liposomes beyond the 30 % obtained above. We previously commented that calcein release is primarily observed during irradiation of the photosensitizer and the generation of ^1^O_2_. It is thus unsurprising that an increase in c22-BMI/r22-*b*-PPO/DPhyPC liposome irradiation time by one hour resulted in an increase in calcein release yield from 30 % to 41 % (Figure 3 a). More fundamentally, we have realized sequence-specific cargo release from two kinds of DBC-unsaturated lipid vesicles composed of either 1,2-dioleoyl-*sn*-glycero-3-phosphocholine (DOPC) or 1,2-dilinolenoyl-*sn*-glycero-3-phosphocholine (DLnPC) lipids, which exhibit two and six double bonds in their hydrophobic tails, respectively (Supporting Information, Scheme S1). We found that DBC-lipid liposomes using DOPC actually show a cargo release effectiveness similar to that of DPhyPC (Figure 3 b). Possibly the presence of only two double bonds in the lipids is insufficient to increase the vulnerability to ^1^O_2_. However, we obtained a marked increase (up to 57 %) in the sequence-specific cargo release from DBC-lipid vesicles based on DLnPC, a clear indication that the inclusion of a significant degree of unsaturation leads to a greater predisposition to membrane damage by ^1^O_2_.

**Figure 3:**
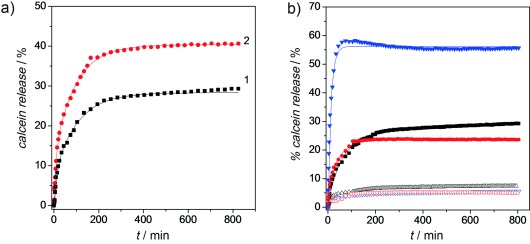
Kinetics of cargo release from DBC-functionalized vesicles induced by ^1^O_2_ formation. a) Effect of irradiation time on calcein release from r22-*b*-PPO/DPhyPC liposomes hybridized with c22-BMI: 104 min (curve 1) and 164 min (curve 2). b) Effect of the phospholipid structure on calcein release from r22-*b*-PPO-coated vesicles hybridized with c22-BMI (solid symbols): DLnPC (blue), DPhyPC (black) and DOPC (red). Hollow symbols show the respective controls (c22-*b*-PPO-coated vesicles in the presence of c22-BMI). Lipid/DBC ratio: 1048.

In conclusion, we have demonstrated that DNA block copolymers are another important class of DNA amphiphiles which can be stably incorporated into the phospholipid bilayer of vesicles, thus tagging these nanocontainers with sequence information. The DNA code on the liposomes was successfully exploited for targeted cargo release. Hybridization of anchored DBCs and a BODIPY monoiodine-ODN photosensitizer conjugate enabled the generation of singlet oxygen close to the lipid membrane by means of light irradiation. The resulting oxidation of the PPO chains or highly unsaturated phospholipids effectively mediates liberation of the vesicle payload. Importantly, we have also confirmed that the cargo release takes place sequence-specifically and only from functionalized containers. As such, we have successfully introduced a novel function of DNA–lipid vesicle systems, and these programmable containers are promising and novel delivery systems that may contribute to substantial improvements and advances in the effectiveness in both the transport and specific release of molecules in nanosystems and devices.

## Notes

Detailed facts of importance to specialist readers are published as ”Supporting Information”. Such documents are peer-reviewed, but not copy-edited or typeset. They are made available as submitted by the authors.
