# Supplementary material for: Light-Triggered Sequence-Specific Cargo Release from DNA Block Copolymer-Lipid Vesicles
Source: Angew Chem Int Ed Engl. 2012 Oct 26;52(3):1008–12. doi: 10.1002/anie.201206783 (PMC3563227; doi:10.1002/anie.201206783)
Supplement: Supplementary file 1 [file anie0052-1008-sd1.pdf]

Angewandte Chemie
Eine Zeitschrift der Gesellschaft Deutscher Chemiker

Supporting Information

© Wiley-VCH 2012

69451 Weinheim, Germany

Light-Triggered Sequence-Specific Cargo Release from DNA Block Copolymer–Lipid Vesicles**

*Alberto Rodríguez-Pulido, Alina I. Kondrachuk, Deepak K. Prusty, Jia Gao, Maria A. Loi, and Andreas Herrmann**

anie_201206783_sm_miscellaneous_information.pdf

Contents

1. Materials -- S2

2. Synthesis protocols -- S3

2.1. Synthesis and characterization of DNA Block Copolymers (DBCs) ----- S3

2.2. Synthesis and characterization of the BODIPY-based photosensitizer -- S8

2.3. Synthesis of ODN-BMI conjugate (c22-BMI) ------------------------------ S9

3. Methods -- S12

3.1. Preparation of DBC-lipid liposomes -- S12

3.2. Dynamic light scattering (DLS) --- S13

3.3. Stable DBC incorporation in liposomes measured by Fluorescence Resonance Energy Transfer (FRET) assay ------------------------------- S14

3.4. Sequence-specific cargo release from liposomes by singlet oxygen generation -- S18

4. Calculations --- S22

5. References --- S24

1. Materials.

Reactants and solvents for DNA and DBC synthesis. Poly(propylene oxide) monobutyl ether (PPO-OH, number average molecular weight M_n = 1000 g/mol), *N,N*-diisopropyl-2-cyanoethyl-chlorophosphoramidite (CEPA-Cl), and *N,N*-diisopropylethylamine (DIPEA) were purchased from Sigma-Aldrich (The Netherlands). Solvents and reagents for DNA synthesis were purchased from Novabiochem (Merck, UK) and SAFC (Sigma-Aldrich, Netherlands). Primer Support™ dC, dG and Universal Primer Support™ (200 µmol/g) purchased from GE Healthcare (Sweden) were used as solid supports during the DNA synthesis. $CDCl_3$, used as the reference for the ^{31}P-NMR spectroscopy, and other chemicals and solvents used in the synthesis and/or chromatography were purchased from Sigma-Aldrich (Germany) and were used without further purification. Distilled water was deionized using a Super Q Millipore system (with a resistivity of 18 MO) to be used in the chemical reactions.

Reactants and solvents for BODIPY monoiodine photosensitizer (BMI) and ODN-BMI conjugate synthesis. All chemicals and reagents were purchased from commercial suppliers and used without further purification, unless otherwise noted. *N*-hydroxy-succinimide (NHS, 98%), dimethylsulfoxide (DMSO) and dimethylformamide (DMF) were purchased from Sigma-Aldrich and were used as received. *N,N'*-dicyclohexyl-carbodiimide (99%) was obtained from Merck. All solvents and reagents for oligonucleotide (ODN) synthesis were purchased from Novabiochem (Merck, UK) and SAFC (Sigma-Aldrich, Netherlands). Solid supports (Primer Support™, 200 µmol/g) from GE Healthcare (Sweden) were used for the synthesis of DNA.

Components and materials for liposomes dispersions. Zwitterionic lipids, 1,2-diphytanoyl-*sn*-glycero-3-phosphocholine (4Me 16:0, DPhyPC), 1,2-dioleoyl-*sn*-glycero-3-phosphocholine (18:1 (? 9-cis), DOPC) and 1,2-dilinolenoyl-*sn*-glycero-3-phosphocholine (18:3 (cis), DLnPC), were purchased from Avanti Polar Lipids (Alabaster, USA) (**Scheme S1a-c**). All of them, with the highest purities (>99%), were used without further purification. Headgroup-labeled phospholipid, lissamine™ rhodamine B 1,2-dihexadecanoyl-*sn*-glycero-3-phosphoethanolamine (triethylammonium salt) (N-Rh-PE, see **Scheme S1d**), was purchased from Invitrogen and used as received. The ODN-dye conjugate r22-Alexa ([5'-CCT CGC TCT GCT

AAT CCT GTT A-3']? [Alexa 488]), the surfactant Triton X-100 (10% in water), the fluorescent dye 2',7'-[bis(carboxymethyl)amino]methyl-fluorescein (calcein) and components of buffer solutions (Tris/HCl buffer) were purchased from Sigma-Aldrich. Anhydrous $CHCl_3$ was purchased from Acros Organics (Belgium) and stored over molecular sieves. The dispersion of liposomes was always performed with doubly deionized water (Super Q Millipore system).

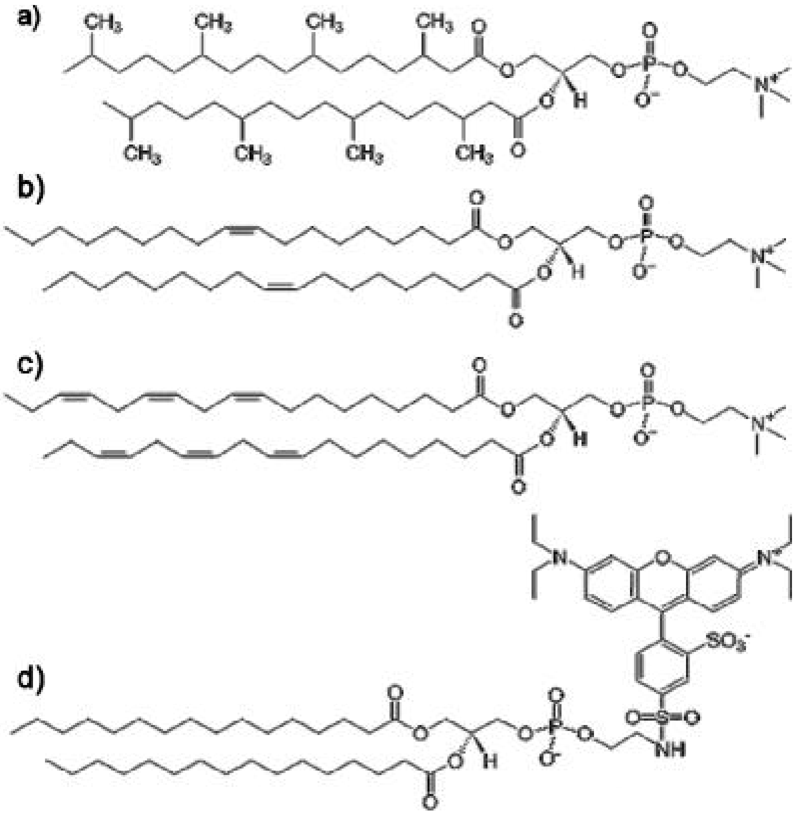

Scheme S1. Structures of the zwitterionic lipids: DPhyPC (**a**), DOPC (**b**) and DLnPC (**c**), and the fluorescent lipid N-Rh-PE (**d**).

2. Synthesis protocols.

2.1. Synthesis and characterization of DNA Block Copolymers (DBCs).

A) Synthesis and characterization of PPO-phosphoramidite. PPO-OH (1.0 mmol) was placed in a round-bottomed flask and dissolved in 25 mL of dry dichloromethane (DCM). CEPA-Cl (2.1 mmol, 500 mg) and DIPEA (2.0 mmol, 350 µL) were added to the polymer solution and then the mixture was stirred at room temperature under argon atmosphere for 2 h. After completion of the reaction, the mixture was dissolved in 100

mL of dry DCM, extracted with 1M Na_2CO_3 solution and washed with water and brine (2×). The solution was dried over $MgSO_4$, filtered and finally, after evaporation of the solvent, the product was dried under high vacuum. The polymer-phosphoramidite was characterized by ^{31}P-NMR spectroscopy and immediately used for the solid phase DNA synthesis. The NMR spectra were recorded on Bruker AMX 400 (400 MHz) spectrometer.

^{31}P-NMR of the PPO-phosphoramidite (400 MHz, $CDCl_3$): 151.3 ppm

B) Synthesis of 22-*b*-PPO and c22-*b*-PPO. The DNA-*b*-PPO DBCs consisting of PPO (1k) coupled to the 5' end of 22 mer ODNs (5'-CCT CGC TCT GCT AAT CCT GTT A-3' for 22-*b*-PPO, and its complementary sequence 5'-TAA CAG GAT TAG CAG AGC GAG G-3' for c22-*b*-PPO), were synthesized using direct solid-phase synthesis as reported previously.[1]

C) Reverse synthesis of r22-*b*-PPO. The DNA-*b*-PPO consisting of PPO coupled to the 3' of 22 mer ODN (5'-CCT CGC TCT GCT AAT CCT A-3') was synthesized with reverse solid-phase synthesis in an ÄKTA Oligopilot 100 plus DNA synthesizer (GE Healthcare, Sweden). The ODN sequence was synthesized in the reverse 5' - 3' direction in 260 µmol scale by a standard phosphoramidite DNA synthesis protocol using a Universal Primer Support (**Scheme S2a**) and the corresponding 3'-DMT-nucleotide-5'-phosphoramidites (**Scheme S2b**). In detail, 1.5 equivalents of the nucleotide phosphoramidite were passed through the column reactor after each detritylation step and were further recycled for 8 min. Once the 22 mer sequence was synthesized, 5

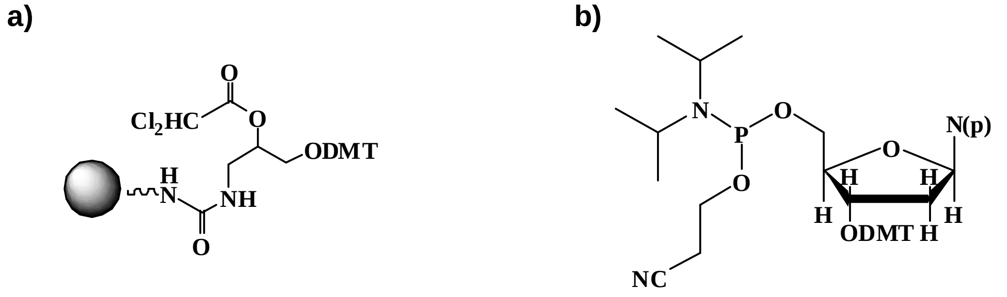

Scheme S2. Structures of reagents used for reverse DNA synthesis: **a)** Universal Primer Support III, PS with linker, and **b)** 3'-DMT-nucleotide-5'-phosphoramidite.

equivalents of PPO-phosphoramidite (0.15 M in dry DCM) mixed with an activator (0.3 M 5-benzylmercaptotetrazole in anhydrous acetonitrile) were passed through the column in order to be coupled with the ODN. The mixture was then recycled for 30 minutes to achieve a higher coupling efficiency. Cleavage of r22-*b*-PPO from the Universal Primer Support was achieved by incubation of the support in 2 M ammonia (methanol solution) at room temperature for 60 minutes. Removal of protective groups from nucleobases was carried out by incubation of the support in 40 mL of concentrated ammonia (aq.) at 60 °C for 16 h. After cooling the ammonia solution to room temperature, the reaction mixture was filtered and the solid support was washed with 1:1 ethanol/water mixture (**Scheme S3**). The filtrate containing DBCs was further purified by anion exchange (AIEX) chromatography.

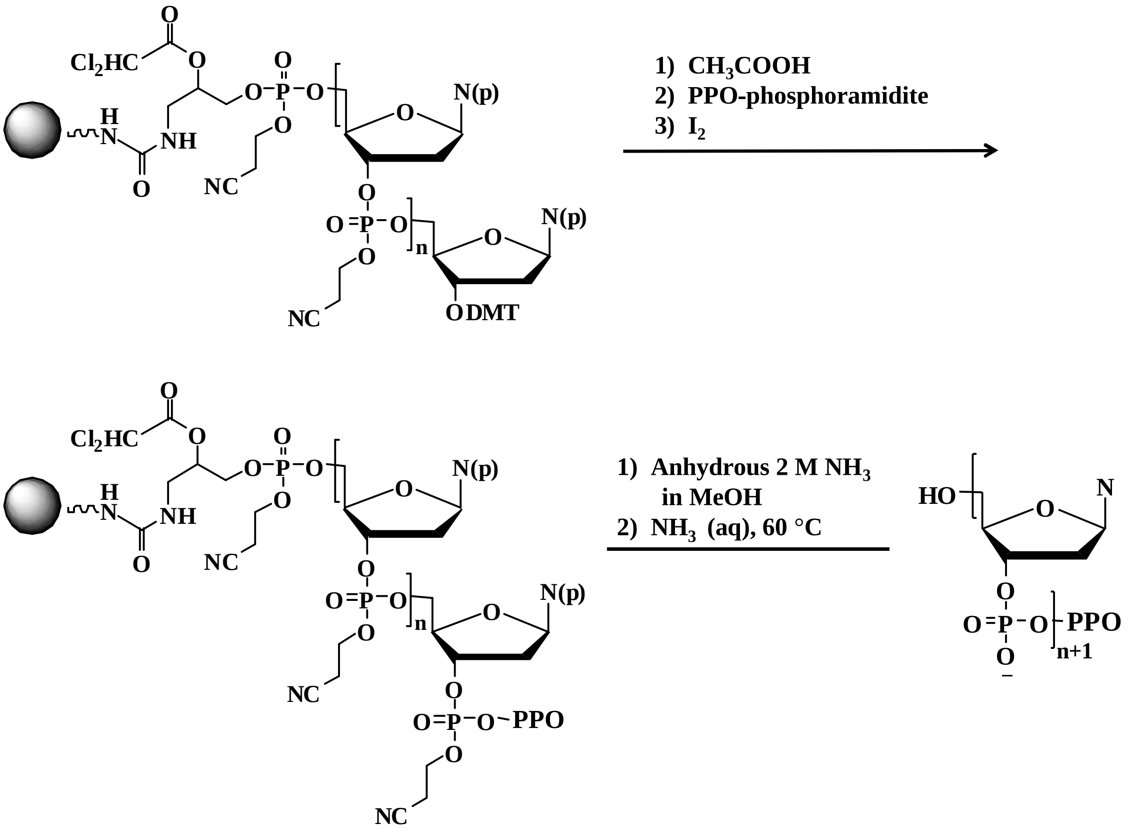

Scheme S3. Synthetic route of r22-*b*-PPO in 5' - 3' direction.

D) Characterization of DBCs 22-*b*-PPO, c22-*b*-PPO and r22-*b*-PPO.

Denaturing Polyacrylamide Gel Electrophoresis (PAGE) analysis. Precast TBE-Urea polyacrylamide (15%) gel (Invitrogen, The Netherlands) was used in the PAGE analysis of the DBCs to confirm the DNA-polymer coupling (**Figure S1**). After electrophoresis, the gel was stained with SYBR Gold nucleic acid gel staining (Invitrogen) and UV transilluminated.

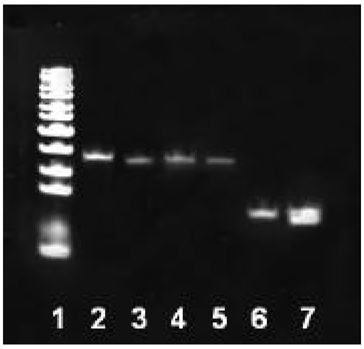

Figure S1. PAGE analysis of purified DBCs. 1: DNA ladder (10 - 300 bp); 2: 22-*b*-PPO; 3: c22-*b*-PPO; 4: cr22-*b*-PPO; 5: r22-*b*-PPO; 6: pristine 22mer DNA with the sequence of 22-*b*-PPO and r22-*b*-PPO; 7: pristine 22 mer DNA with the sequence of c22-*b*-PPO.

Mass Spectrometry. Molecular weights of the synthesized DBCs were determined using matrix-assisted laser desorption/ionization time of flight MALDI-TOF mass spectrometry. The mass spectra (**Figure S2**) were recorded on a Bruker MALDI-TOF mass spectrometer. The following matrix was employed: 20 mg of 3-hydroxypicolinic acid, 2.0 mg picolinic acid, 3.0 mg ammonium citrate, 0.5 ml of a mixture of ultra pure water/acetonitrile (7:3); ratio sample: matrix = 1:2 (v/v). The concentration of the DNA solution was 100 µM.

Anion Exchange (AIEX) Chromatography. Analytical AIEX Chromatography was performed using a HiTrap Q HP column (GE Healthcare, 5 mL column volume) on ÄKTA Purifier (GE Healthcare, UK) with in-line multi-wavelength UV-Vis detector. Two elution buffers were used (A: 25 mM Tris-HCl, pH = 8.0 and B: 25 mM Tris-HCl, pH = 8.0, 1.0 M NaCl) through a linear gradient. UV absorbance at 254 nm was monitored during the elution (**Figure S3**).

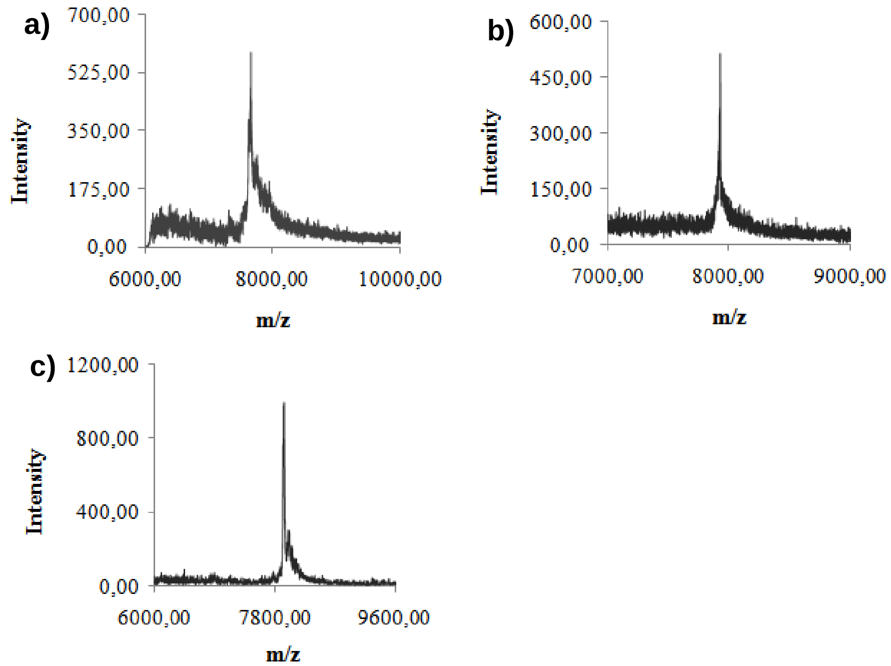

Figure S2. MALDI-TOF spectra of DNA-*b*-PPO. **a)** 22-*b*-PPO (found: 7700 m/z, calculated: 7680 g/mol); **b)** c22-*b*-PPO (found: 7970 m/z, calculated: 7920 g/mol); and **c)** r22-*b*-PPO (found: 7940 m/z, calculated: 7920 g/mol).

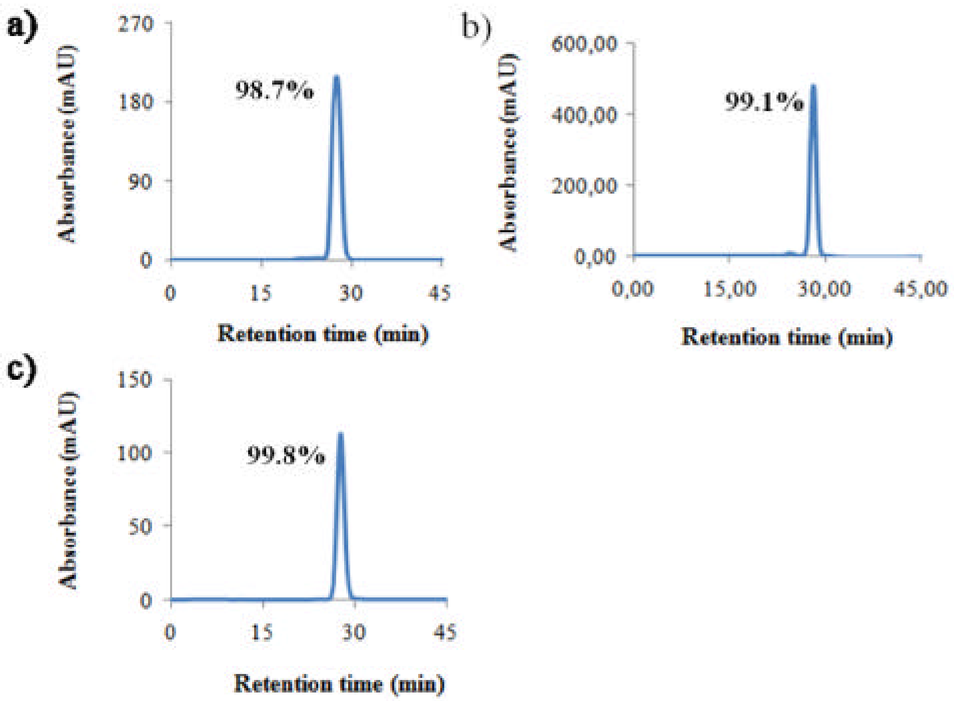

Figure S3. AIEX elution graphs of the purified products: r22-*b*-PPO (**a**), 22-*b*-PPO (**b**), and c22-*b*-PPO (**c**). Numbers beside the elution peak represent the percentage of product area versus total area (purity) integrated from the chromatogram.

2.2. Synthesis and characterization of BODIPY-based photosensitizer.

In order to release encapsulated compounds from DBC-lipid vesicles, with light as a trigger, a dye that catalyzes the formation of singlet oxygen was utilized. By the action of this species the vesicle membrane is destabilized and concomitantly the cargo released.

For that purpose we employed a BODIPY dye that was recently developed in our laboratory in the context of DNA-templated fluorogenic reactions.[2] The dye consists of a bora-indacene scaffold which is functionalized with carboxylic acid and iodine groups (**Figure S4**). The synthesis of this compound was reported elsewhere.[2] The carboxylic acid functionality was utilized for the conjugation to amino-modified ODNs while the iodine group induced intersystem crossing resulting from the heavy atom substituent.

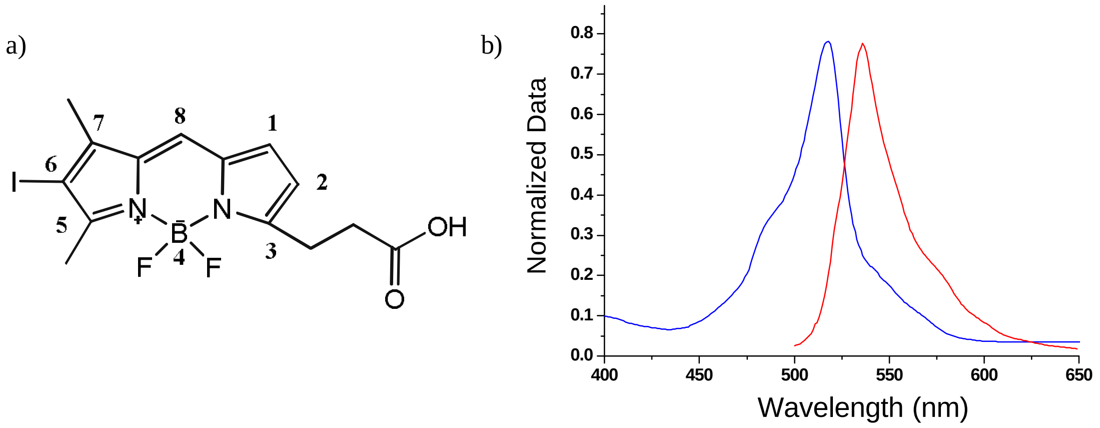

Figure S4. a) Chemical structure of the BODIPY monoiodine (BMI) photosensitizer. **b**) Normalized absorption (blue) and emission (red) spectra of BMI ($?_{ab,max}$ = 527 nm; $?_{em,max}$ = 536 nm) in chloroform at 25 °C. The fluorescence quantum yield of BMI (f_{fl} = 0.03) was determined in chloroform against the reference cresyl violate in methanol at 25 °C, indicating efficient intersystem crossing.

It is well-known that the introduction of iodine group into chromophores favors triplet formation through inter-system crossing.[3] To check the generation of singlet oxygen, the steady-state photoluminescence of singlet oxygen was measured, as described in the literature.[4] A $5\times10^{?5}$ M BODIPY monoiodine (BMI) methanol solution was excited with a 150 fs pulsed Kerr mode-locked Ti-sapphire laser

(Coherent, USA), with doubled frequency at 380 nm and 8 mW output power. The singlet oxygen emission spectrum was recorded at room temperature with an InGaAs detector (Andor Technology, USA) calibrated for the instrumental response. **Figure S5** shows the steady-state photoluminescence spectrum of singlet oxygen originated from BMI triplet population in comparison with the signal obtained from a common standard for singlet oxygen generation, Rose Bengal, usually utilized for photodynamic therapy. The experiment proves that our BMI dye is excellently suited for the purpose of singlet oxygen generation and is even 14 times more efficient than Rose Bengal.

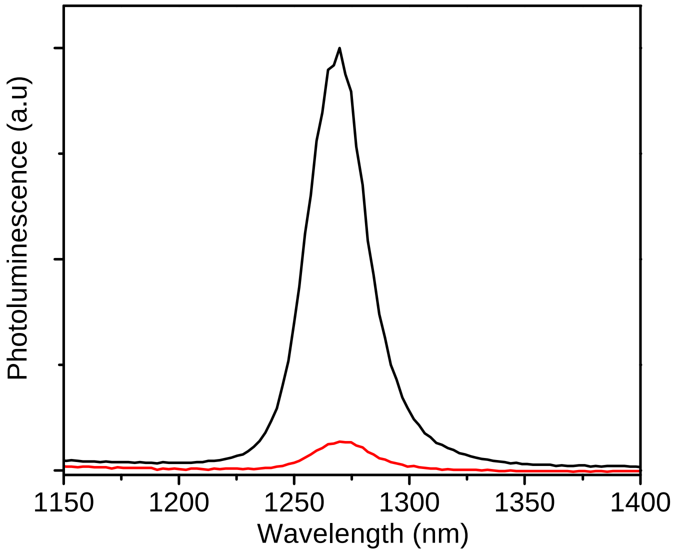

Figure S5. Near-Infrared singlet oxygen photoluminescence spectra from BMI (black) and Rose Bengal (red) in MeOH ($5\times10^{?5}$ M) excited by a Ti-sapphire laser at 380 nm with 8 mW output power.

2.3. Synthesis of ODN-labeled BMI conjugate (c22-BMI).

A) Materials and methods. An amino-modified oligonucleotide (ODN) was synthesized using standard automated solid-phase phosphoramidite coupling methods on an ÄKTA oligopilot plus (GE Healthcare) DNA synthesizer. The ODN was purified by reverse-phase High Pressure Liquid Chromatography (HPLC) using a C15 RESOURCE RPCTM 1 mL reverse phase column (GE Healthcare) through custom gradients using elution buffers (A: 100 mM triethylammonium acetate (TEAAc) and 2.5% acetonitrile, B: 100 mM TEAAc and 65% acetonitrile). Fractions were further desalted by either desalting column (HiTrapTM desalting, GE Healthcare) or dialysis

membrane (MWCO 2000, Spectrum Laboratories). The labeled ODN was purified by HPLC and characterized by MALDI-TOF mass spectrometry using a 3-hydroxypicolinic acid matrix. The spectrum was recorded on an ABI Voyager DE-PRO MALDI-TOF (delayed extraction reflector) Biospectrometry Workstation mass spectrometer. Absorption spectra were measured on a SpectraMax M2 spectrophotometer (Molecular Devices, USA) using an 1 cm light-path quartz cell.

B) Synthesis of NHS ester of the mono-iodo BODIPY dye. The carboxyl group of mono-iodo BODIPY propionic acid was activated by reacting BMI (0.046 g, 0.11 mmol) with *N*-hydroxysuccinimide (NHS) (0.036 g, 0.30 mmol) and *N*,*N'*-dicyclohexyl-carbodiimide (DCC) (0.037 g, 0.32 mmol) in 1 mL of DMF. The reaction was carried out for 24 h under inert atmosphere at room temperature (**Scheme S4**). Precipitated dicyclohexylurea (DCU) was removed by filtration. The solvent was evaporated under reduced pressure and the crude mixture was purified by chromatography using hexane/EtOAc (1:1) as eluent. Activated product **2** was obtained as a violet solid (31 mg, 58%).

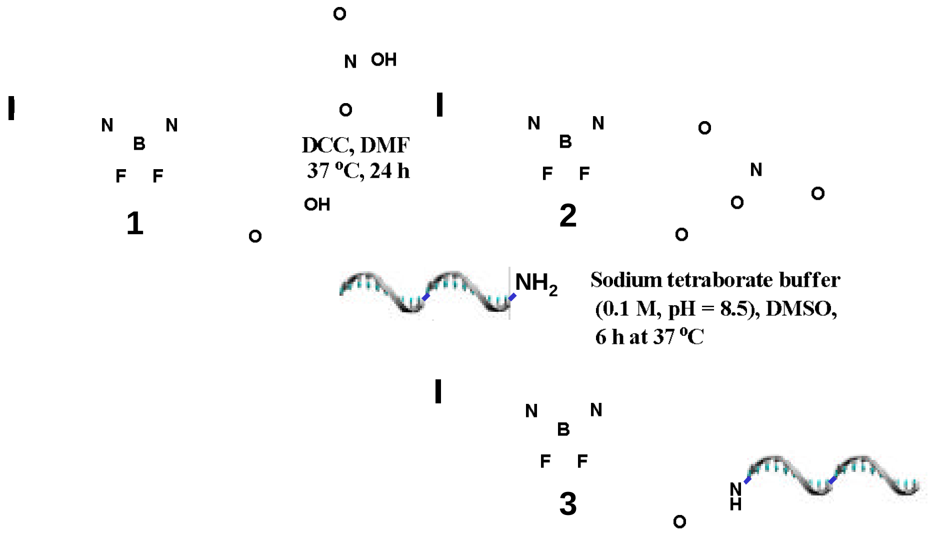

Scheme S4. Schematic representation of the synthetic route of BODIPY-labeled ODN (c22-BMI).

C) Synthesis and characterization of c22-BMI: DNA labeling with mono-iodo BODIPY NHS ester. 22mer 5′-(C6) amino-modified ODN (5'-TAA CAG GAT TAG CAG AGC GAG G-3') was dissolved in sodium tetraborate buffer (0.1 M, pH = 8.5) at

a concentration of 1 nmol/µL. 100 µL of this ODN solution was mixed with a solution of activated BODIPY NHS ester **2** in DMSO (20 µL, 40 µg/µL). The resulting reaction mixture was mixed in a shaker for 24 h at room temperature and subsequently freeze-dried to remove the DMSO and H_2O (**Scheme S4**). Purification of the labeled ODN was carried out by using reverse-phase HPLC employing a C15 RESOURCE RPC™ 1 mL column (GE Healthcare) and custom gradients with elution buffers (A: 100 mM TEAAc and 2.5% acetonitrile, B: 100 mM TEAAc and 65% acetonitrile). The coupling yield of the labeling reaction was estimated to be 50% from the integration of the peaks of the HPLC chromatogram. The purified BODIPY-labeled ODN (i.e. c22-BMI, peak at ~ 35 mL (**Figure S6a**) was analyzed by UV-Vis absorption spectroscopy (**Figure 6b**) and MALDI-TOF mass spectrometry (**Figure 7**).

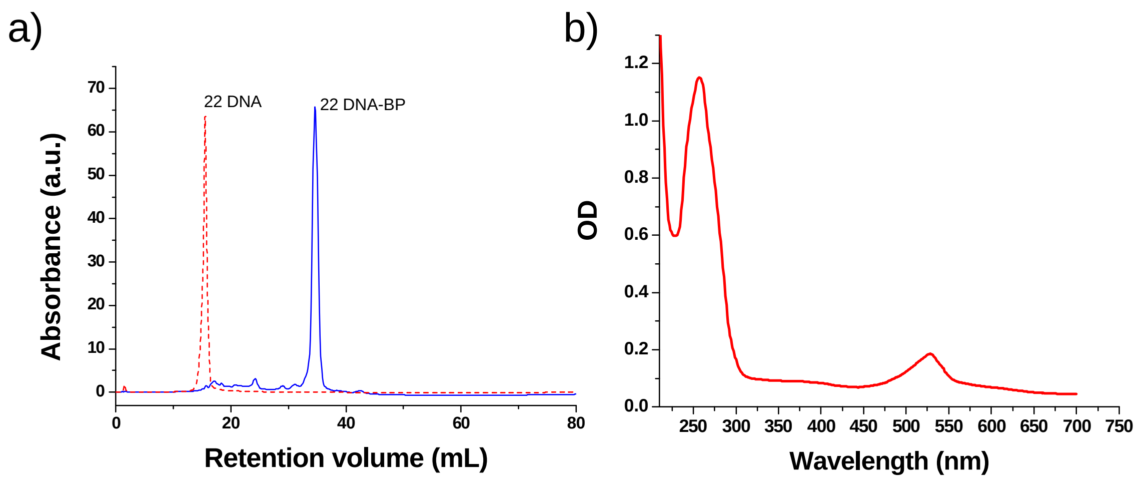

Figure S6: (**a**) Reverse phase HPLC chromatogram of c22-BMI after purification (blue)**.** Elution was monitored at 260 nm. This material was analyzed by UV/Vis spectroscopy. The dashed red line represents a control, i.e. amino-modified ODN starting material. (**b**) Absorption spectrum of the purified c22-BMI**.** Peaks at 260 nm and 527 nm correspond to ODN and BODIPY absorbance, respectively.

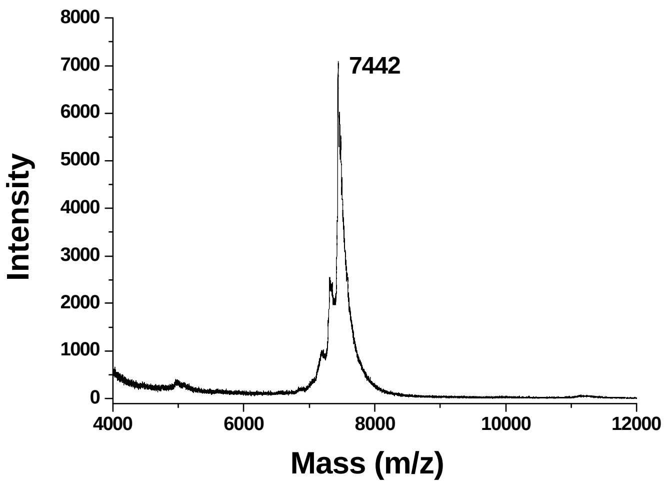

Figure S7: MALDI-TOF mass spectrum of BODIPY-labeled 22ODN (c22-BMI). Calculated: 7435 m/z; found 7442 m/z.

3. Methods.

3.1. Preparation of DBC-lipid liposomes.

The DBC-lipid liposome formation was based on a protocol described previously for lipid vesicles,[5] with some modifications. Typically an appropriate amount of freeze-dried DNA-*b*-PPO was mixed with a pertinent volume of lipid in chloroform, to reach the required lipid:DBC ratio. Afterwards the organic solvent was removed by evaporation under a nitrogen stream and then under vacuum overnight. The resulting dry DBC-lipid films were hydrated with an aqueous buffer: a) 10 mM Tris/HCl buffer, pH = 7.4, 150 mM NaCl, in experiments to prove the stable incorporation of DBCs in liposomes, or b) 10 mM Tris/HCl buffer, pH = 7.4, containing 100 mM calcein, in experiments to observe the cargo release from liposomes by singlet oxygen formation. In both buffers the lipid concentration was 0.4 mg/mL. A combination of vortexing and small periods of sonication helped with hydration and yielded a polydisperse population of multilamellar vesicles (MLVs). The MLV dispersions were extruded through a polycarbonate membrane (Whatman, pore size 200 nm) to form large unilamellar vesicles, LUVs, of higher homogeneity, by using a Thermobarrel Lipex Extruder (Northern Lipids). DBC-lipid liposomes were never used for more than one day.

Liposomes with encapsulated calcein were separated from non-encapsulated probes by the use of Sephadex G-75 size exclusion columns (GE Healthcare) equilibrated with 10 mM Tris/HCl, pH = 7.4, 150 mM NaCl buffer. Osmolality was confirmed to be the same before and after chromatography. The phospholipid concentration after the size exclusion column was determined by the well-known Stewart assay based on colorimetric complex formation ($?_{ab,max}$ = 485 nm) between ammonium ferrothiocyanate and the phospholipids.[6] Sample concentrations were determined as the mean of three independent measurements.

When DLnPC lipid was used, small changes in the protocol were introduced to avoid the premature oxidation of its six double bonds (see **Scheme S1**). To create the dry DLnPC:DBC film, the evaporation of chloroform took place under an argon stream and then under vacuum for no more than 30 min. Degassed buffers were used to hydrate the film and also to equilibrate the size exclusion column.

3.2. Dynamic light scattering (DLS).

DLS was carried out to measure the mean liposome diameters before and after the size exclusion column. The measurements were performed with an ALV (CGS-3), working in autocorrelation mode and using the red line (*?* = 632.8 nm) of a helium-neon laser (JDSU 1145/P). An average correlation function was obtained for each sample from at least 10 measurements at a fixed temperature of 25.0 °C and a scattering angle, *?*, of 90°, corresponding to the wavevector, $q = (4pn/?)\sin(?/2) = 1.87 \times 10^5$ cm^{-1}, where n is the solution refractive index. The normalized second-order correlation functions, $g^{(2)}(t)$, were analyzed using CONTIN[7] inverse Laplace algorithm. From the average relaxation times, t, the apparent diffusion coefficients, D_{app}, were obtained, and using the Stokes- Einstein relation,[8] the apparent hydrodynamic radii, $R_{h,app}$, were calculated:

$$\frac{1}{t} = D_{app} q^2 = \frac{k_B T}{6ph R_{h,app}} q^2 \qquad [1]$$

As an example, **Figure S8** shows the normalized correlation functions and the relaxation time distributions obtained for 22-*b*-PPO/DPhyPC and c22-*b*-PPO/DPhyPC liposomes. The mean of diameters was measured after extrusion to be 196 ± 20 nm for all DBC-lipid liposomes used here. No appreciable change in the mean size and/or size

distributions was observed between different lipids or DBCs, including after size exclusion chromatography.

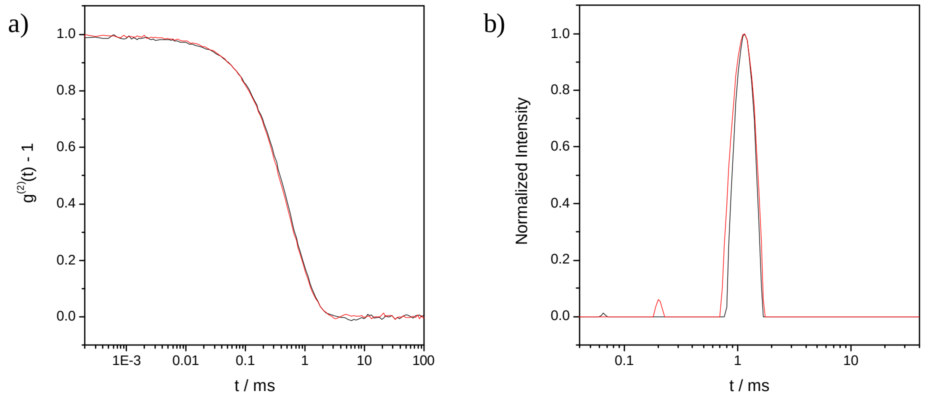

Figure S8. Normalized correlation functions (**a**) and relaxation time distributions (**b**) at a scattering angle ? = 90° for 22-*b*-PPO/DPhyPC liposomes (black) and c22-*b*-PPO/DPhyPC liposomes (red) at ratio lipid/DBC = 1048.

3.3. Stable DBC incorporation in liposomes by Fluorescence Resonance Energy Transfer (FRET) assay.

Alexa 488 and rhodamine dyes show energy transfer when there is a sufficiently short distance between them, as reported previously.[9] Fluorescence emission spectra of the pair r22-Alexa (donor) and N-Rh-PE (acceptor) in the 500–700 nm region were recorded with excitation at 470 nm using a Cary Eclipse (Varian, Australia) fluorescence spectrophotometer. Measurements were carried out at constant temperature of 25.0 °C with a reflowing water circuit. In all cases, excitation and emission band slits were fixed at 2.5 nm and a 10 mm light-path quartz cell was used.

A) Formation of FRET system. An effective FRET system, consisting of r22-Alexa/c22-*b*-PPO/N-Rh-PE/DPhyPC liposomes (**Scheme S5a**), was created to study the stability of DBC incorporation into the membranes. In detail, c22-*b*-PPO was incorporated in N-Rh-PE/DPhyPC (3:97 mol/mol) liposomes as explained above, to obtain DBC-lipid liposomes at the ratio lipid:DBC 370:1. Then an aliquot of these liposomes was mixed with a small amount of r22-Alexa such that [r22-Alexa] = [c22-*b*-PPO] = 0.972 µM and with a final lipid concentration of 0.361 mM. Subsequently, c22-

PPO and r22-Alexa were hybridized with an Eppendorf Mastercycler (Germany). The optimized protocol consisted of heating the mixture 15 min to 40 °C and slowly cooling to 4 °C for 140 min. Afterwards the emission spectra of r22-Alexa/N-Rh-PE pair were taken as explained above. The disappearance of the FRET process was confirmed by disrupting the FRET liposomes following addition of Triton X-100 at a final concentration of 0.1% (v/v).

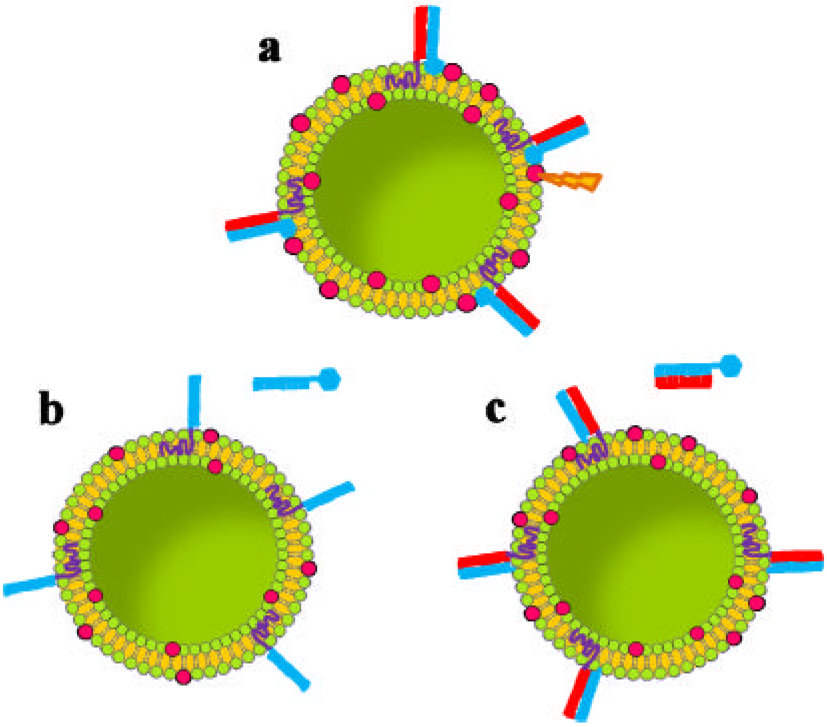

Scheme S5. Schematic pictures of FRET system (**a**) and non-FRET systems (**b** and **c**, controls) fabricated to measure the stable DBC incorporation into lipid membranes: **a**) r22-Alexa/c22-*b*-PPO/N-Rh-PE/DPhyPC liposomes, **b**) 22-*b*-PPO/N-Rh-PE/DPhyPC liposomes mixed with r22-Alexa and **c**) ds-22-*b*-PPO/N-Rh-PE/DPhyPC liposomes mixed with ds-22-Alexa.

B) Formation of non-FRET systems (controls). The "energy transfer vesicles" of the preceding DBC-lipid liposome system were compared with two control experiments in which FRET did not occur: 22-*b*-PPO/N-Rh-PE/DPhyPC liposomes mixed with r22-Alexa (**Scheme S5b**), and ds-22-*b*-PPO/N-Rh-PE/DPhyPC liposomes mixed with ds-22-Alexa (**Scheme S5c**). DBC-lipid liposomes were prepared at the same concentrations as in the FRET system ([DPhyPC] = 0.361 mM and [22PPO] = 0.972 µM). To prepare system **5b**, 22-*b*-PPO/N-Rh-PE/DPhyPC liposomes were mixed with an aliquot of 0.972 µM r22-Alexa and the sample was subjected to the previous

hybridization protocol to perform the same experimental conditions. However, hybridization was not possible because ss-ODNs of r22-Alexa and 22-*b*-PPO have the same sequence. On the other hand, sample **5c** was prepared by separately hybridizing solutions of c22-*b*-PPO/N-Rh-PE/DPhyPC liposomes and r22-Alexa, both with their complementary ODN 22mers (22 and c22, respectively). Once double strands were created, ds-22-*b*-PPO/N-Rh-PE/DPhyPC liposomes were mixed with ds-22-Alexa at a final ratio of 22-*b*-PPO:r22-Alexa 1:1. The fluorescence spectra of r22-Alexa/N-Rh-PE pair of **5b** and **5c** systems were comparable to the spectrum obtained from the FRET system after disrupting it with Triton X-100.

C) Assay to determine the stability of the DBCs in the liposome membranes over time. FRET liposomes consisting of r22-Alexa/c22-*b*-PPO/N-Rh-PE/DPhyPC were mixed with non-fluorescent (NF) liposomes (prepared exclusively from pure DPhyPC) at different FRET:NF v/v ratios: 1:1, 1:10 and 1:100, respectively (**Scheme S6**). The final lipid concentration was kept constant at [DPhyPC] = 0.361 mM in the three resulting samples. The r22-Alexa/N-Rh-PE emission spectra of each ratio were taken as a function of the time, until 24 h after mixing.

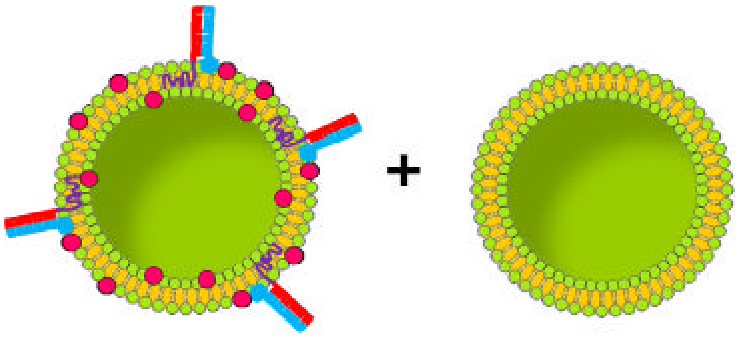

Scheme S6. Schematic picture of the stability measurements of DBCs in liposomes overtime: r22-Alexa/c22-*b*-PPO/N-Rh-PE/DPhyPC FRET liposomes (left) are mixed with non-fluorescent (NF) DPhyPC liposomes (right) at different FRET:NF ratios (1:1, 1:10 and 1:100).

D) Detection limit of the FRET experiment. The detection limit of the FRET experiment was determined by comparing the r22-Alexa/N-Rh-PE spectra between FRET systems (r22-Alexa/c22-*b*-PPO/N-Rh-PE/DPhyPC liposomes, **Scheme S5a**) and non-FRET systems (22-*b*-PPO/N-Rh-PE/DPhyPC liposomes mixed with r22-Alexa, **Scheme S5b**), at different DBC concentrations. The detection limit corresponds to the

DBC concentration at which it is not possible to detect the difference between FRET and NF spectra. In all sets of FRET and non-FRET systems the final r22-Alexa and DPhyPC/N-Rh-PE (97:3) concentrations were kept constant at 0.972 µM and 0.361 mM, respectively, and the DBC (c22-*b*-PPO or 22-*b*-PPO) concentrations ranged from 0.039 to 0.972 µM. **Figure S9a** shows the r22-Alexa/N-Rh-PE fluorescence spectra taken for FRET and non-FRET systems for all DBC concentrations studied. As can be seen in the figure, a lower DBC concentration in the lipid membrane yields a smaller difference between FRET and non-FRET spectra. **Table S1** contains the values of r22-Alexa/N-Rh-PE fluorescence intensity ratios, I_{590}/I_{520}, from the spectra obtained previously and **Figure S9b** shows a plot of I_{590}/I_{520} as a function of lipid:DBC ratio for FRET and non-FRET systems. The figure allows a determination of the detection limit of the fluorescence technique as the intersection of the trendlines for FRET and no-FRET systems, roughly at lipid:DBC ratio of 6000:1. This limit can be conveniently transformed into DBCs/liposome by **Equation S3** (see section 4), giving rise to ~ 48 DBCs per liposome, which is significantly smaller than the ratio used in the experiment (~ 775 DBCs per liposome).

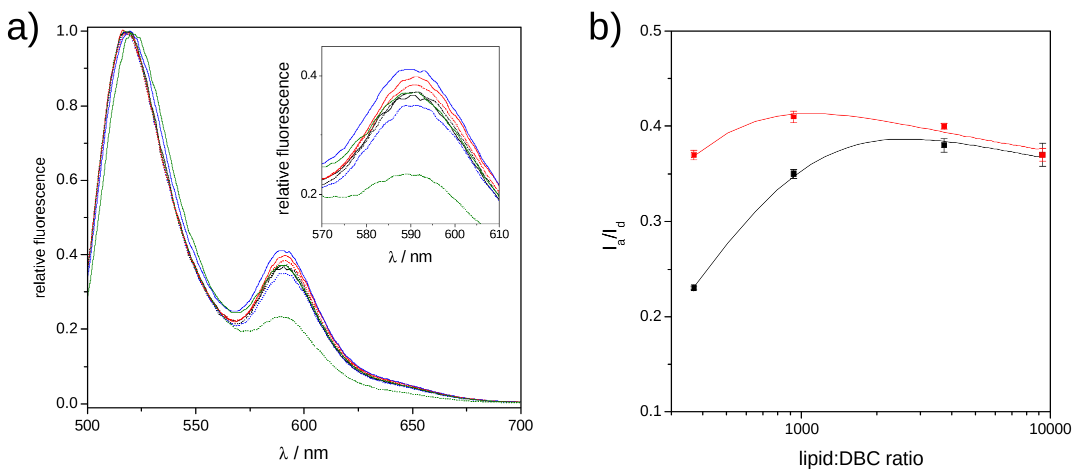

Figure S9. a) r22-Alexa/N-Rh-PE fluorescence spectra of FRET liposomes (r22-Alexa/c22-*b*-PPO/N-Rh-PE/DPhyPC liposomes, solid lines) and non-FRET liposomes (22-*b*-PPO/N-Rh-PE/DPhyPC liposomes mixed with r22-Alexa, dotted line) at different DBC:lipid ratios: 370, green; 930, blue; 3750, red; and 9300, black. The inset shows a zoom-in of the acceptor N-Rh-PE band. **b**) Plot of N-Rh-PE/22-Alexa fluorescence intensity ratios, I_{590}/I_{520}, from FRET (red) and non-FRET (black) spectra.

Table S1. Lipid:DBC and DBC:liposome ratios used for determining the detection limit of the FRET assay, and the acceptor/donor maxima fluorescence intensity ratios, I_{590}/I_{520}.

Lipid:DBC ratio	DBC:liposome ratio[a]	I_{590}/I_{520} FRET system[b]	I_{590}/I_{520} non-FRET system[c]
9300	31	0.37	0.37
3750	78	0.40	0.38
930	308	0.41	0.35
370	775	0.37	0.23

[a] DBC:liposome ratio was determined by the Equation S3. [b] FRET system: r22-Alexa/c22-*b*-PPO/N-Rh-PE/DPhyPC liposomes. [c] Non-FRET system: 22-*b*-PPO/N-Rh-PE/DPhyPC liposomes mixed with r22-Alexa.

3.4. Sequence-specific cargo release from liposomes by singlet oxygen generation.

Singlet oxygen was generated by irradiating the c22-BMI molecule at a wavelength of 530 nm using the xenon flash lamp (50 Watts, 3 flash/s) of a SpectraMax M2 spectrophotometer (Molecular Devices, USA). The singlet oxygen generation generally took place over 104 min, but this time was increased to 164 min when the effect of irradiation time was analyzed. The cargo release from the DBC-lipid vesicles was studied by monitoring the maximum fluorescence intensity of calcein. This probe can be easily contained in the vesicles and passes through the membrane only when a pore or leak is formed. Usually, the dye is incorporated at high concentrations (higher than 80 mM) at which it is self-quenched. However, the release gives rise to dilution, such that the increase in the fluorescence intensity can be easily monitored.[9] Moreover, the absorption and emission maxima of calcein (485/510 nm) make it the ideal dye for the release experiments described herein due to the fact that this wavelength region does not interfere with the absorption spectrum of the photosensitizer c22-BMI. In this way, the cargo release can be monitored without affecting singlet oxygen formation. ~~This well-known method is based on the increase of the calcein fluorescence intensity that takes place upon dilution of the initially self-quenching molecules.[10]~~ Fluorescence measurements were carried out at a temperature of 25.0 °C for around 15 h in the same spectrophotometer during and after irradiation. A 10 mm light-path quartz cell was used in the experiments.

A) Method. r22-*b*-PPO/DPhyPC, r22-*b*-PPO/DOPC and r22-*b*-PPO/DLnPC liposomes with encapsulated calcein (100 mM) were created at a lipid concentration of 0.080 mM. To observe the cargo release as a function of the DBC concentration in the membrane, the lipid:DBC ratio was varied from 1048:1 to 9270:1. 100 µL of the resulting liposomal dispersions were mixed with a small aliquot of 2 µM c22-BMI solution, and then r22-*b*-PPO and c22-BMI were hybridized using an Eppendorf Mastercycler (Germany). The optimized protocol consisted of heating the mixture 15 min to 40 °C and slowly cooling to 4 °C for 140 min. Afterwards the dispersions were placed in a quartz cell and were irradiated at a wavelength of 530 nm. The excitation of c22-BMI generated singlet oxygen close to the lipid membranes which induced calcein leakage from the vesicles. The dilution effect in the calcein concentration led to an increase of calcein fluorescence, which was continuously monitored at the maximum emission intensity (?$_{em}$ = 510 nm) by excitation at 485 nm. To determine the fluorescence intensity maximum corresponding to complete release of calcein, an aliquot of Triton X-100 was added at the end of the recording time to a final concentration of 0.1% (v/v). The percentage of calcein release was calculated as:

$$\%\ release = \frac{I_t - I_0}{I_\infty - I_0} \times 100 \qquad [2]$$

where I_0 is the initial fluorescence intensity of calcein-loaded liposomes (before c22-BMI irradiation), I_t is the fluorescence intensity during and after irradiation time and I_8 corresponds to the fluorescence intensity after the complete destruction of liposomes by the addition of Triton X-100 aqueous solution.

Two control samples were studied in order to ensure that liposomes were releasing the cargo due to the effect of the singlet oxygen: 1) r22-*b*-PPO/DPhyPC liposomes without the presence of the photosensitizer, c22-BMI, and 2) c22-*b*-PPO/DPhyPC liposomes in the presence of c22-BMI (note that DBC and photosensitizer have the same ODN sequence). Both controls were created at a lipid:DBC ratio of 1048:1, i.e. the highest DBC concentration in the membranes. Samples were prepared by following the same protocol described above and at the equivalent final lipid concentration. Although no photosensitizer was present in control **1**, it was irradiated under the same conditions in order to observe the effect of UV-Vis irradiation on the DBC-lipid liposomes. The low percentage of calcein release obtained (~ 6%) reveals that DBC-lipid liposomes are scarcely affected by direct irradiation. On the other hand, the

photosensitizer as well as the singlet oxygen generated in control **2** stay in the bulk, sufficiently far from the vesicles, due to the fact that hybridization with the DBC is impossible. The long average distance between the vesicles and the singlet oxygen generated in the bulk should ensure that the membrane is not strongly affected and thus that calcein is not released. This supposition is confirmed by the fact that control **2** shows a low % of release (~ 7%), very similar to the release of control **1**, in which DBC-lipid liposomes were only subjected to irradiation.

B) Degradation of DBC by singlet oxygen. A native polyacrylamide gel electrophoresis (PAGE) analysis was performed in order to investigate the photo-degradation of r22-*b*-PPO by singlet oxygen. DBCs consisting of r22-*b*-PPO were hybridized with photosensitizer-labeled complementary ODN (c22-BMI) by following a similar hybridization protocol as described above. Hybridization products were separated from non-hybridized compounds by carrying out AIEX chromatography. The purification was performed using a HiTrap Q HP column (GE Healthcare, 5 mL column volume) on an ÄKTA Purifier (GE Healthcare) supplied with an in-line multi-wavelength UV-Vis detector, utilizing a custom gradient 0 - 1 M NaCl. UV absorbance at 254 nm was monitored during the elution. Samples of purified r22-*b*-PPO/c22-BMI were then irradiated for 2, 4, 6 and 8 hours. Irradiated and non-irradiated samples were loaded in a precast 20% polyacrylamide TBE electrophoresis gel (Invitrogen, The Netherlands). After electrophoresis the gel was stained with SYBR Gold nucleic acid gel staining (Invitrogen) and UV transilluminated at 260 nm. **Figure S10a** shows that r22-*b*-PPO/c22-BMI (lane 6) is susceptible to degradation. Already after 2 hours of irradiation the main band was fading and a smear with higher electrophoretic mobility appeared. This behavior hints towards the formation of oxidation products and scission. After 8 h irradiation time almost all r22-*b*-PPO/c22-BMI was degraded into low molecular weight products (lane 10).

With the aim to discern if oxidative damage takes place at the DNA sequence or at the polymeric chain, another PAGE assay was realized. Pristine 22 mer DNA was hybridized with c22-BMI and separated from non-hybridized compounds by following the same protocols as previously mentioned. The purified double stranded photosensitizer labeled complementary ODN (ds-22-BMI) was then irradiated for 2, 4, 6 and 8 hours, respectively, and submitted to PAGE analysis. **Figure S10b** shows that the irradiation of ds-22-BMI (lane 4) gives rise to the disappearance of its main band in

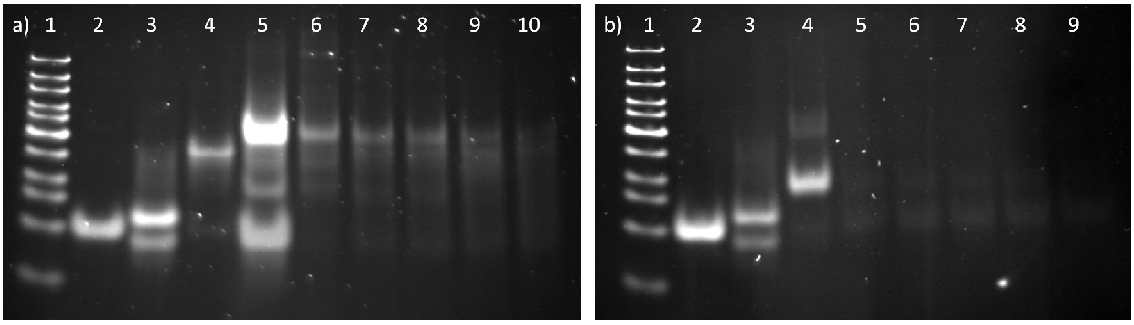

Figure S10. a) PAGE analysis of effect of light irradiation on DBCs hybridized with BMI. 1: DNA ladder (10 - 300 bp); 2: pristine c22 mer DNA; 3: c22-BMI; 4: r22-*b*-PPO; 5: r22-*b*-PPO/c22 hybridization products; 6: r22-*b*-PPO/c22-BMI hybridization products; 7 to 10: r22-*b*-PPO/c22-BMI irradiated for 2, 4, 6 and 8 hours, respectively. **b)** PAGE analysis of effect of light irradiation on pristine DNA hybridized with BMI. 1: DNA ladder (10 - 300 bp); 2: pristine c22 mer DNA; 3: c22-BMI; 4: ds22-BMI; 5 to 9: ds-22-BMI irradiated for 2, 4, 6 and 8 hours, respectively.

expense to the appearance of a smeared band with higher electrophoretic mobility (lane 5 to 9), indicating oxidative damage of DNA to a considerable extent.

Due to the fact that singlet oxygen is involved in the formation of hydroxyl radicals which have high oxidative effect on the polymer,[11] we have studied the oxidation of the pristine PPO polymer chain present in DBCs using FT-IR spectroscopy. A mixture of BMI and pure PPO dissolved in $CHCl_3$ was irradiated at 530 nm for 2, 4 and 8 hours, respectively. Then irradiated and non-irradiated samples were placed on a Nicolet 8700 Nexus FT-IR spectrometer (Thermo Scientific Inc., USA) and IR spectra were taken as an average of 16 measurements (**Figure S11**). The figure shows an increase in the carbonyl absorption peak (1724 cm^{-1}) when the PPO/BMI mixture is irradiated (solid lines), as compared with irradiated samples in absence of the polymer (dotted lines). This result indicates that PPO is susceptible to oxidation by singlet oxygen and/or hydroxyl radicals in our systems. It is anticipated that the appearance of carboxylic acid and hydroperoxyde groups change the polarity of the polymeric hydrophobic chain of DBCs, such that the lipid bilayer is destabilized allowing the cargo release.

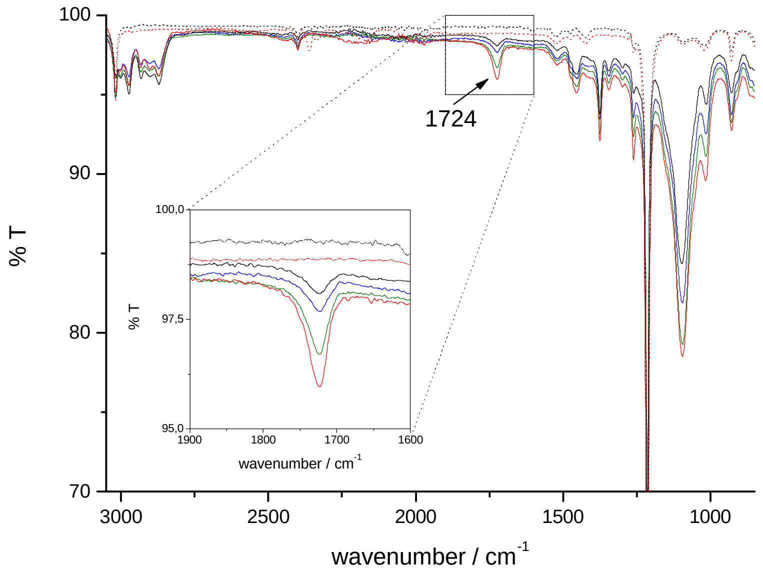

Figure S11. FT-IR spectra of a mixture of PPO and BMI in $CHCl_3$ (solid black) and its oxidation products after irradiating the mixture at 530 nm for 2, 4 and 8 hours (solid blue, solid green and solid red lines, respectively). Dotted lines represent the FT-IR spectra of BMI in $CHCl_3$ before and after irradiation for 4 hours (black and red lines, respectively). The inset shows in detail the carbonyl absorption peak located at 1724 cm^{-1}.

4. Calculations.

4.1. DBC:liposome ratio determination.

Lipid/DBC ratios, determined by the concentrations of both components, have been conveniently transformed into the number of DBCs per liposome (DBC/liposome ratio) by using the equation

$$DBC / liposome = \frac{\Phi}{(lipid / DBC)} \qquad [3]$$

where F is the number of lipids per liposome which can be calculated from geometrical considerations:

$$\Phi = \frac{20p\left(0.1d^2 - d + 5\right)}{a_0} \qquad [4]$$

where the numerator is the surface area of the spherical DBC-lipid liposomes with averaged radius d, and a_0 is the reported[12] surface area of the lipid hydrophilic head (=

0.8 nm^2). For the calculations, we have assumed a thickness of the lipid bilayer of approximately 5 nm width[8,13] and an average radius similar to the average hydrodynamic radius determined by DLS (d = D_{app}).

4.2. Range of singlet oxygen and its effect on liposomes.

To estimate the diffusion of the singlet oxygen in buffer medium, statistical thermodynamic assumptions have been taken into account. The maximum distance of singlet oxygen travelling in solution can be determined from the root-mean-square linear displacement, $d_{½}$:

$$d_{½} = \left(2tD\right)^{1/2} \qquad [5]$$

where t is twice the lifetime of singlet oxygen (t = 2 × t; t = 3.1 µs)[14] and D its diffusion coefficient. Assuming a diffusion coefficient of oxygen in pure water[15] of 2 × 10$^{?\,5}$ cm^2 s$^{?\,1}$ the calculation gives a range for the singlet oxygen of $d_{½}$ = 157 nm.

On the other hand, we have determined the average distance between two DBC-lipid liposomes for our specific experimental case from geometrical considerations. Assuming static spherical liposomes (their diffusion coefficient is ~ 1000 times higher than that of the oxygen) with a simple cubic packing factor in buffer medium (i.e. one liposome per unit cell), we can define the average distance between vesicles, $d_{\text{ves-ves}}$, as:

$$d_{\text{ves-ves}} = \left(\frac{\Phi}{N_{\mathrm{A}} L}\right)^{1/3} - d \qquad [6]$$

where F was defined previously in equation S4, N_{A} is the Avogadro constant, L is the final lipid concentration and d is the average diameter of the DBC-lipid liposomes (d = D_{app}). Equation S6 gives a distance between liposomes of $d_{\text{ves-ves}}$ = 1860 nm, which is one order of magnitude larger than the calculated displacement of singlet oxygen $d_{½}$. This rationalizes our assumption that singlet oxygen only induces cargo release when produced close to the vesicle membrane, that is only the case when the ODN-photosensitizer is hybridized to the DBC incorporated in the membrane.

5. References

[1] a) F. E. Alemdaroglu, K. Ding, R. Berger, A. Herrmann, *Angew. Chem. Int. Ed.* **2006**, *45*, 4206-4210; b) M. Anaya, M. Kwak, A. J. Musser, K. Müllen, A. Herrmann, *Chem. Eur. J.* **2010**, *16*, 12852-12859.

[2] D. K. Prusty, A. Herrmann, *J. Am. Chem. Soc.* **2010**, *132*, 12197-12199.

[3] T. Yogo, Y. Urano, Y. Ishitsuka, F. Maniwa, T. Nagano, *J. Am. Chem. Soc.* **2005**, *127*, 12162-12163.

[4] a) H. H. Wasserman, R. W. Murray, *Singlet Oxygen*, Academic Press, Inc., New York, **1979**; b) A. A. Krasnovsky Jr, *Biofizica* **1976**, *21*, 748-749; c) A. U. Khan, M. Kasha, *Proc. Natl. Acad. Sci. USA* **1979**, *76*, 6047-6049.

[5] a) A. Rodriguez-Pulido, E. Aicart, O. Llorca, E. Junquera, *J. Phys. Chem. B* **2008**, *112*, 2187-2197; b) A. Rodriguez-Pulido, F. Ortega, O. Llorca, E. Aicart, E. Junquera, *J. Phys. Chem. B* **2008**, *112*, 12555-12565.

[6] J. C. M. Stewart, *Anal. Biochem.* **1980**, *104*, 10-14.

[7] a) S. W. Provencher, *Comput. Phys. Commun.* **1982**, *27*, 213-227; b) S. W. Provencher, *Comput. Phys. Commun.* **1982**, *27*, 229-242.

[8] D. R. Lide, *CRC Handbook of Chemistry and Physics*, CRC Press, Boca Raton, **2004**.

[9] Y. Muto, Y. Okano, in *Current Issues on Multidisciplinary Microscopy Research and Education* (Eds.: A. Méndez-Vilas, L. Labajos-Broncano), Formatex, Badajoz, **2006**, pp. 39-44.

[10] a) T. M. Allen, L. G. Cleland, *Biochim. Biophys. Acta* **1980**, *597*, 418-426; b) C. E. Kundrot, E. A. Spangler, D. A. Kendall, R. C. Macdonald, R. I. Macdonald, *Proc. Natl. Acad. Sci. USA* **1983**, *80*, 1608-1612.

[11] a) F. Posada, J. L. Philippart, P. Kappler, J. L. Gardette, *Polym. Degrad. Stab.* **1995**, *50*, 141-158; b) L. Costa, G. Camino, M. P. Luda, G. G. Cameron, M. Y. Qureshi, *Polym. Degrad. Stab.* **1996**, *53*, 301-310; c) M. Kitis, C. D. Adams, G. T. Daigger, *Water Res.* **1999**, *33*, 2561-2568.

[12] a) D. Marsh, *CRC Handbook of Lipid Bilayers*, CRC Press, Boca Raton, **1990**; b) C. M. Wiethoff, M. L. Gill, G. S. Koe, J. G. Koe, C. R. Middaugh, *J. Biol. Chem.* **2002**, *277*, 44980-44987.

[13] a) D. Attwood, A. T. Florence, *Surfactant Systems: Their Chemistry, Pharmacy and Biology*, Chapman and Hall, London, **1983**; b) J. H. Fendler, *Membrane Mimetic Chemistry*, John Wiley & Sons, New York, **1982**; c) C. Tanford, *The Hydrophobic Effect: Formation of Micelles and Biological Membranes*, John Wiley & Sons, New York, **1980**.

[14] a) S. Y. Egorov, V. F. Kamalov, N. I. Koroteev, A. A. Krasnovsky Jr, B. N. Toleutaev, S. V. Zinukov, *Chem. Phys. Lett.* **1989**, *163*, 421-424; b) C. Schweitzer, R. Schmidt, *Chem. Rev.* **2003**, *103*, 1685-1757.

[15] a) M. Tsushima, K. Tokuda, T. Ohsaka, *Anal. Chem.* **1994**, *66*, 4551-4556; b) I. Zebger, J. W. Snyder, L. K. Andersen, L. Poulsen, Z. Gao, J. D. C. Lambert, U. Kristiansen, P. R. Ogilby, *Photochem. Photobiol.* **2004**, *79*, 319-322.
